# Sedentary Behavior Research Network (SBRN) – Terminology Consensus Project process and outcome

**DOI:** 10.1186/s12966-017-0525-8

**Published:** 2017-06-10

**Authors:** Mark S. Tremblay, Salomé Aubert, Joel D. Barnes, Travis J. Saunders, Valerie Carson, Amy E. Latimer-Cheung, Sebastien F.M. Chastin, Teatske M. Altenburg, Mai J.M. Chinapaw, on behalf of SBRN Terminology Consensus Project Participants, Teatske M. Altenburg, Saeideh Aminian, Lauren Arundell, Andrew J. Atkin, Salomé Aubert, Joel Barnes, Bethany Barone Gibbs, Rebecca Bassett-Gunter, Kevin Belanger, Stuart Biddle, Aviroop Biswas, Valerie Carson, Jean-Philippe Chaput, Sebastien Chastin, Josephine Chau, Mai ChinAPaw, Rachel Colley, Tara Coppinger, Catharine Craven, Carlos Cristi-Montero, Douglas de Assis Teles Santos, Borja del Pozo Cruz, Jesus del Pozo-Cruz, Paddy Dempsey, Ricardo Filipe do Carmo Santos Gonçalves, Ulf Ekelund, Laura Ellingson, Victor Ezeugwu, Claire Fitzsimons, Alberto Florez-Pregonero, Ciarán P. Friel, Andreas Fröberg, Lora Giangregorio, Linda Godin, Katie Gunnell, Shannon Halloway, Trina Hinkley, Jill Hnatiuk, Pauliina Husu, Mohammad Kadir, Leonidas G. Karagounis, Annemarie Koster, Jeroen Lakerveld, Martin Lamb, Richard Larouche, Amy Latimer-Cheung, Allana G. LeBlanc, Eun-Young Lee, Paul Lee, Luis Lopes, Trish Manns, Taru Manyanga, Kathleen Martin Ginis, Joanne McVeigh, Joilson Meneguci, Carla Moreira, Elaine Murtagh, Freda Patterson, Danilo Rodrigues Pereira da Silva, Arto J. Pesola, Neil Peterson, Cherie Pettitt, Lara Pilutti, Snehal Pinto Pereira, Veronica Poitras, Stephanie Prince, Apoorva Rathod, Fabien Rivière, Sara Rosenkranz, François Routhier, Rute Santos, Travis Saunders, Brett Smith, Olga Theou, Jennifer Tomasone, Mark Tremblay, Patricia Tucker, Renée Umstattd Meyer, Hidde van der Ploeg, Tania Villalobos, Toni Viren, Birgit Wallmann-Sperlich, Katrien Wijndaele, Roderick Wondergem

**Affiliations:** 1 Healthy Active Living and Obesity Research Group, Children’s Hospital of Eastern Ontario Research Institute, 401 Smyth Road, Ottawa, ON K1H 8L1 Canada; 2 Department of Applied Human Sciences, University of Prince Edward Island, Charlottetown, PEI C1A 4P3 Canada; 3 Faculty of Physical Education and Recreation, University of Alberta, Edmonton, AB T6G 2H9 Canada; 4 School of Kinesiology and Health Studies, Queen’s University, Kingston, ON K7L 3N6 Canada; 5 Institute of Applied Health Research, Glasgow Caledonian University, Glasgow, Scotland; 6 Department of Movement and Sport Sciences, Ghent University, Ghent, Belgium; 7 VU University Medical Center, Amsterdam Public Health Research Institute, Amsterdam, Netherlands

**Keywords:** Physical inactivity, Sedentary behavior, Stationary behavior, Standing, Screen time, Non-screen-based time, Sitting, Reclining, Lying, Bouts, Interruptions, Breaks

## Abstract

**Background:**

The prominence of sedentary behavior research in health science has grown rapidly. With this growth there is increasing urgency for clear, common and accepted terminology and definitions. Such standardization is difficult to achieve, especially across multi-disciplinary researchers, practitioners, and industries. The Sedentary Behavior Research Network (SBRN) undertook a Terminology Consensus Project to address this need.

**Method:**

First, a literature review was completed to identify key terms in sedentary behavior research. These key terms were then reviewed and modified by a Steering Committee formed by SBRN. Next, SBRN members were invited to contribute to this project and interested participants reviewed and provided feedback on the proposed list of terms and draft definitions through an online survey. Finally, a conceptual model and consensus definitions (including caveats and examples for all age groups and functional abilities) were finalized based on the feedback received from the 87 SBRN member participants who responded to the original invitation and survey.

**Results:**

Consensus definitions for the terms physical inactivity, stationary behavior, sedentary behavior, standing, screen time, non-screen-based sedentary time, sitting, reclining, lying, sedentary behavior pattern, as well as how the terms bouts, breaks, and interruptions should be used in this context are provided.

**Conclusion:**

It is hoped that the definitions resulting from this comprehensive, transparent, and broad-based participatory process will result in standardized terminology that is widely supported and adopted, thereby advancing future research, interventions, policies, and practices related to sedentary behaviors.

**Electronic supplementary material:**

The online version of this article (doi:10.1186/s12966-017-0525-8) contains supplementary material, which is available to authorized users.

## Background

There has been rapid and progressive growth in research studying sedentary time and sedentary behaviors [1–8]. Increasing evidence of the link between excessive sedentary behavior and adverse health indicators or outcomes has perpetuated this interest [3, 5–7, 9–15]. The American Heart Association recently issued a science advisory on sedentary behavior and cardiovascular morbidity and mortality [16]. As this field of research has grown, so too has confusion over the definition of sedentary behavior (see Table 1 for a sample of definitions) and other related terms (e.g., screen time, sedentary behavior patterns, bouts, breaks) [4, 7, 17–22]. For example, Altenburg and Chinapaw [17] recently recommended that to advance the field of sedentary behavior epidemiology, standardized procedures for accelerometer data collection and data reduction should be defined; standardized operational definitions of sedentary bouts and breaks are needed; sedentary bouts should be operationally defined as a minimum period of uninterrupted sedentary time; total time accumulated in sedentary bouts per day (or per week) should be calculated rather than using average bout duration; and the best mathematical adjustment that converts accelerometer data into sedentary patterns should be further explored. The majority of sedentary behavior self- and proxy-report questionnaires in the published literature have not undergone psychometric testing [10]. Of those that have undergone testing, a small number have acceptable levels of reliability, although the validity of most questionnaires is low [23]. Several other large-scale projects are currently underway to produce a common taxonomy, harmonized measurement protocols, consistent data reduction and analytic procedures, and a framework for establishing research priorities (Table 2). However, a collaborative global initiative to arrive at consensus definitions on key terms has remained elusive.

**Table 1:** Sample of definitions of sedentary behavior from the research literature

Definition	Reference
“Sedentary behavior may be defined as having a MET value between one and 1.5 (for example, equivalent to sitting or lying down)”.	[65]
“Sedentary behaviors were defined as having MET <2.0 (e.g., equivalent to sitting or lying down)”.	[66]
“A distinct class of behaviors characterized by low energy expenditure”.	[67]
“Sedentary behavior involves activities with a very low energy expenditure (1.0–1.8 metabolic equivalents [MET]), performed mainly in a sitting or supine position”.	[68]
“Sedentary behavior refers to activities that do not increase energy expenditure substantially above the resting level and includes activities such as sleeping, sitting, lying down, and watching television, and other forms of screen-based entertainment. Operationally, sedentary behavior includes activities that involve energy expenditure at the level of 1.0–1.5 metabolic equivalent units (METs)”.	[4]
“Sedentary behaviors such TV viewing, computer use, or sitting in an automobile typically are in the energy-expenditure range of 1.0 to 1.5 METs (multiples of the basal metabolic rate). Thus, sedentary behaviors are those that involve sitting and low levels of energy expenditure”.	[2]
“Sitting, lying down, and expending very little energy (approximately 1.0–1.5 metabolic equivalents [METs])”.	[56]
“Non-upright” activities”.	[69]
“Sedentary behaviours are considered those requiring ≤1.5 METs.”	[7]
“Sedentary behaviour, defined as time spent sitting or lying”.	[70]
“The term sedentary behavior (from the Latin word sedere, “to sit”) describes a distinct class of activities that require low levels of energy expenditure in the range of 1.0–1.5 METs (multiples of the basal metabolic rate) and involve sitting during commuting, in the workplace and the domestic environment, and during leisure”.	[6]
“Any waking behavior characterized by energy expenditure ≤1.5 metabolic equivalents (METs) while in a sitting or reclining posture”.	[25]

**Table 2:** Initiatives related to, or possibly beneficiaries of, the SBRN Terminology Consensus Project

AlPHABET	The AlPHABET project is an open science project set up to develop a common taxonomy (naming and cataloging system) for classification, harmonization and storage of objective tracking sensor data of human physical behavior in daily life [71]. Development will be through an international consensus process using the Delphi method. It aims to reach international consensus on an overarching definition for the study of how activities, physical actions and movements as part of human daily behavior impacts health and well-being; and on an integrated classification system, data model and nomenclature. A brief description is available online [71].
International Society for the Measurement of Physical Behavior	The International Society for the Measurement of Physical Behavior is a non-profit scientific society which focuses on issues related to ambulatory monitoring, wearable monitors, movement sensors, physical activity, sedentary behavior, movement behavior, body postures, sleep and constructs related to physical behaviors. It aims to promote and facilitate the study and applications of objective measurement and quantification of free-living physical behavior using wearable devices. More information about this society, its membership, actions or shared resources can be found on its website [72].
International Society for Physical Activity and Health	The International Society for Physical Activity and Health (ISPAH) [73] has a mission to advance and promote physical activity as a global health priority through excellence in research, education, capacity building and advocacy. The ISPAH has recently launched a Sedentary Behavior Council [74] to specifically focus on advancing science, advocacy, and practice related to sedentary behaviour.
Sensor Methods Collaboratory (National Institutes of Health)	The Sensor Methods Collaboratory is an initiative created after a pre-conference workshop at the 3rd International Conference on Ambulatory Monitoring of Physical Activity and Movement (ICAMPAM) in June 2013 which was held to propose a collaborative approach to algorithm development to interpret ambulatory monitoring of movement behaviours [75]. Researchers would benefit if a commonly accepted approach to data reduction can ultimately be achieved. The collaboration aims to provide an opportunity for the research community to discuss needs, develop shared resources, propose standard protocols and metadata requirements, pilot new tools, and disseminate methodological research for further evaluation or implementation. At this time, smaller working groups are being formed to propose solutions to different issues (ethics and privacy; harmonizing calibration protocols and data elements; hardware, software and data management logistics; and evaluating evidence and comparison of analytic approaches) and efficient models for dissemination of the results of these deliberations will be explored [76].
Sedentary Behavior International Taxonomy (SIT)	SIT is an open science project setup to develop a common taxonomy of sedentary behaviors through a formal consensus process taking into account the opinion of experts and of the general public. The first round of the Delphi method involved experts who were asked to make statements about the taxonomy; its purpose and use; the domains, categories or facets that should be considered and included; the structure/architecture to arrange and link these domains and facets has been reported [77]. The SIT taxonomy aims to facilitate systematic and standardised investigation and analysis, to enable systematic and standardised reporting, to facilitate comparison and meta-analysis and to facilitate development of measurement tools, sensors and outcome measures of sedentary behaviors. Contributions from interested researchers remain welcomed; further information can be found online [78].
Sedentary Behaviour Research Network (SBRN)	SBRN is a network of researchers and practitioners interested in the health-impact of sedentary behaviour. SBRN’s mission is to connect sedentary behaviour researchers and health professionals working in all fields of study, and to disseminate this research to the academic community and to the public at large. Further information can be found online [24].
Systems of Sedentary Behaviors (SOS) Framework	The SOS-framework is an international transdisciplinary consensus framework developed for the study of determinants, research priorities and policy on sedentary behavior across the life course [79]. A comprehensive concept mapping approach was used to develop this framework, involving an international expert scientist working group which was recruited directly based on publication records in the field of sedentary behavior research, their respective field of expertise and focus on specific stage of the life course. The final framework consisted of six clusters of determinants: Physical Health and Wellbeing, Social and Cultural Context, Built and Natural Environment, Psychology and Behavior, Politics and Economics, and Institutional and Home Settings. The framework can be used as a tool to prioritize future research and to develop policies to reduce sedentary time.

In 2012, the Sedentary Behaviour Research Network (SBRN; a network connecting sedentary behavior researchers and health professionals from around the world interested in sedentary behavior research [24]) published a letter proposing definitions aimed at clarifying differences between “sedentary behaviour” and “physical inactivity” [25]. This clarification, authored by 52 SBRN members, achieved wide acceptance and was published in English [25, 26] and was subsequently translated and published in French [27], Portuguese and Spanish [28]. There remains, however, a need for further refinement and consensus on a variety of related and emergent terms (e.g., screen time, standing, sitting, reclining), and it has become clear that some commonly used terms are ill-suited for specific populations, such as young children (e.g., before they can sit or stand), those with chronic disease (e.g., hyperthyroidism), or those with mobility impairment (e.g., where postural challenges with standing may represent light or moderate physical activity). Furthermore, the standardized use of key terms (e.g., sedentary behavior vs physical inactivity) has had variable uptake across disciplines (e.g., behavioral science vs exercise physiology) and medical subject headings (MESH) continue to use physical inactivity when sedentary behavior would be more appropriate. Collectively, these issues underscore the need for standardization of terminology, and for definitions that have utility across all ages and physical abilities.

Building on previous work, the SBRN orchestrated a comprehensive effort to further develop consensus definitions for terms related to sedentary behavior research, for all age groups and for all physical abilities, through engagement of its membership. The purpose of this paper is to report on the process employed, conceptual model created, and consensus definitions developed for terms routinely used in research related to sedentary behavior.

## Methods

**Fig. 1:**
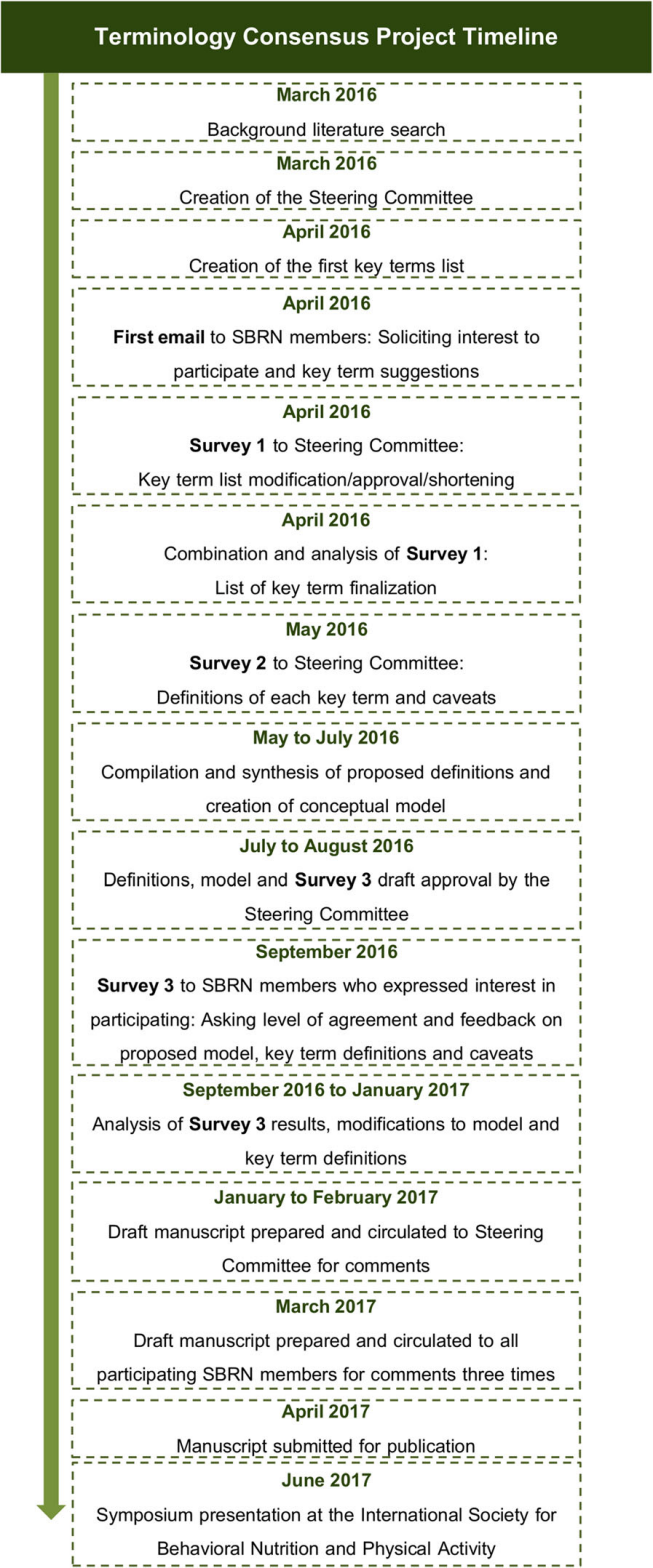
Terminology Consensus Project timeline

A series of sequential processes were employed in an effort to derive consensus definitions for key terms in sedentary behavior research (Fig. 1). These processes included i) a literature search, ii) establishment of a Steering Committee of SBRN members, iii) invitation to all SBRN members to participate in the consensus project, iv) selecting a list of key terms, v) developing a conceptual model, vi) drafting definitions for key terms, vii) collecting input and feedback on the conceptual model and draft definitions from participating SBRN members, viii) compiling input and finalizing (reaching consensus on) the conceptual model and definitions, ix) preparing the manuscript with sign-off by all participants, x) and finally disseminating project results through publication and presentation.

A literature search was performed in March 2016 to i) review the current use of the SBRN [25] definition, and potential deviations from this definition, and ii) examine current operational definitions of sedentary behavior and related terms (e.g., screen time, sedentary behavior patterns, bouts and breaks) and any evidence of inconsistencies, differences, conflicts, or concerns over variations in definitions employed. To identify current relevant articles a search with filters capturing papers published in the past 5 years (May 2011 to May 2016) was conducted in PubMed (see Table 3 for search terms). The articles were selected if there was mention of sedentary behavior and/or related term definitions, cut-points, measurement challenges/recommendations or standard processing techniques for accelerometer data in the title or abstract, with no regard to the population (e.g., child or adult; healthy or unhealthy), type of study, intervention, or comparison being explored. After reviewing the included full-text articles, data were extracted from relevant articles including the aim of the study, defined/discussed terms, targeted population, and definitions/relevant information.

**Table 3:** Literature search strategy

((definition*[Title/Abstract] OR consensus[Title/Abstract] OR standard*[Title/Abstract]) AND
(sedentary[Title/Abstract] OR sitting[Title/Abstract] OR reclining[Title/Abstract] OR
stationary[Title/Abstract])) AND (time[Title/Abstract] OR duration*[Title/Abstract] OR
bout*[Title/Abstract] OR pattern*[Title/Abstract] OR interruption*[Title/Abstract] OR
break*[Title/Abstract] OR type*[Title/Abstract] OR characteristic*[Title/Abstract] OR intermittence*[Title/Abstract])

In addition to gathering background information for the project, the literature search allowed for the identification of authors of key papers who were invited to form a Steering Committee for the SBRN Terminology Consensus Project (MST (Chair), TJS, VC, AEL, SFMC, TA, MJMC and project management support from SA and JDB from SBRN). Key terms from the literature search were collated and the Steering Committee members were asked to add or remove key terms from the list. A final draft list of terms was agreed upon by this Committee. An email was sent to the SBRN membership, consisting of researchers, scholars, practitioners, trainees and students interested in sedentary behavior (1094 members worldwide in April, 2016), soliciting interest in participating in the project and asking for suggestions for key terms to be included in the survey.

The Steering Committee identified the most common key terms reported and deliberated through a short survey and email communication to arrive at draft definitions for each term, important caveats for certain age and ability groups, examples to assist with interpretation and, when available, references for the proposed definition. The final list of terms included stationary behavior, sedentary behavior, standing, screen time, non-screen-based sedentary time, sitting, reclining, lying, and sedentary behavior pattern. This process also led to the development of a conceptual model to help position the key terms in relation to one another. The draft definitions, caveats, examples, references, and conceptual model were included in a survey developed for distribution to participating SBRN members. The survey asked participants to assess the clarity of, and agreement with, the conceptual model and proposed definitions (using a five point scale from strongly agree to strongly disagree), while also providing an opportunity for general comments. Consensus was determined a priori to have been achieved if ≥75% of respondents strongly agreed or somewhat agreed with a particular question (see Additional file 1: for complete survey). Note that the term “physical inactivity” was not included in the survey because there were no suggested changes to the existing SBRN [25] definition.

Input from all participants, including the Steering Committee, was consolidated (by MST, SA, JDB) and revisions to the conceptual model, definitions, caveats, examples, and references were made by the Steering Committee. The draft manuscript, including the revised conceptual model and definitions, was sent to all participants for additional review and comments. After additional revisions, a revised draft of the manuscript was resent to all participants for comments, group consensus, and assessment of likelihood of use. Finally, the further revised manuscript (third review) was recirculated to the project participant group for final comments and sign-off for submission.

## Results

### Literature review

The literature search identified 997 articles, and after screening for inclusion criteria, 14 were used for the identification and definition of key terms in sedentary behavior research. The definition of sedentary behavior as “any waking behaviour characterized by an energy expenditure ≤1.5 METs while in a sitting or reclining posture,” proposed by SBRN [25] was widely adopted as evidenced by frequent citations (558 according to SCOPUS, March 20, 2017), though at least one article claimed that there is still not a real consensus on the definition [20]. Support for the SBRN definition was found for adults [29] and children [21].

The SBRN definition has dual components, including both energy expenditure and posture. The postural aspect is easily operationalized and widely used to identify sedentary behavior using questionnaires, direct observation or inclinometers, but tends to ignore the energetic component. In contrast, the energetic aspect, often determined by indirect calorimetry or accelerometry, has received criticism in relation to its inability to discriminate between postures. However, it should be noted that accelerometers assess movement rather than energy expenditure, and therefore at best represent an indirect method of assessing energy expenditure in any population.

Mansoubi et al. [29] concluded that the 1.5 metabolic equivalent (MET) intensity threshold used in the SBRN definition of sedentary behavior was generally appropriate for distinguishing between common sitting and standing activities in healthy weight and obese adults; however, some common sitting behaviors (e.g., typing) sometimes have a MET level above this threshold. In a recent study that compared MET-defined cut-points used to classify sedentary behaviors in children aged 7–13 years in simulated free-living conditions, it was reported that adult MET thresholds may not be appropriate for children and that the upper limit of the MET threshold for sedentary behavior in children should be increased from 1.5 to 2.0 METs [22]. These authors suggested that a more precise, age-specific definition of sedentary behavior in children may improve overall physical activity (mis)classification in the population and may be of value in quantifying the health burden of sedentary behaviors in youth. In contrast, using whole-room calorimetry as the criterion measure, Reilly et al. [21] and others [30] concluded that the current MET threshold recommendation of ≤1.5 METs seems appropriate for young children (mean age of 5.3 years). Other research supports the inclusion of postural condition (“while in a sitting or reclining posture”) in the sedentary behavior definition instead of just using a definition based on energy cost (≤1.5 METs) [31, 32]. Despite a few inconsistencies, in general, there was broad support in the published literature for the SBRN [25] definition of sedentary behavior, at least in individuals without mobility impairment.

The SBRN [25] definition has also been used for individuals with mobility impairment, for example in studies of people with multiple sclerosis [33–35] and community-dwelling older adults (≥60 years of age) who self-reported mobility impairments [36]. The cut-point of ≤1.5 METs was accepted and used for these populations to distinguish between sedentary behavior and light physical activity. There is much debate concerning how to measure sedentary behavior with accelerometers in individuals with different mobility impairments. While the threshold of ≤100 cpm is the most commonly used cut-point [35], Ezeugwu and colleagues [33] suggested that it may actually represent light intensity activity for some people with mobility impairment and that there is still a need for further research to determine appropriate accelerometer cut-points among groups with different mobility impairments.

Altenburg and colleagues [18] examined the occurrence and duration of sedentary bouts, and explored the cross-sectional associations of various operational definitions of sedentary bouts with health indicators in children. Their study highlighted the potential influence of varying definitions of a sedentary bout or sedentary break on their association with health indicators, and provided support for the need for a consensus definition of these terms. Altenburg and Chinapaw [17] discussed and proposed definitions of sedentary bouts and breaks in studies among children. They recommended that a sedentary bout should be defined as a minimum period of uninterrupted sedentary time, not allowing any “tolerance time” (e.g., time spent in non-sedentary behaviors). They further recommended calculating the total time spent in sedentary bouts (per day or per week) instead of average bout duration and they proposed that a sedentary break be defined as a non-sedentary period in between two sedentary bouts. Based on analyses of associations between accelerometer-derived sedentary bout length and cardiovascular disease risk factors using the U.S. National Health and Nutrition Examination Survey, Kim et al. [37] proposed that a threshold of 5 or 10 min be used to define a sedentary bout.

The literature search gathered evidence supporting, and related to, the standardization, harmonization and consensus initiatives summarized in Table 2. For example, several articles highlighted the need for evidence-based and standardized accelerometer cut-points, data collection protocols and data reduction procedures for assessing sedentary time in children and adults [19, 38–43]. These are important areas for future work but beyond the scope of this Terminology Consensus Project.

#### SBRN participant survey findings

The Terminology Consensus Project survey included an overarching conceptual model and draft definitions of key terms identified through the literature search and from project participants. The complete survey (i.e., survey 3 in Fig. 1) sent to respondents is provided in Additional file 1. The survey was organized to be consistent with the hierarchical structure of the conceptual model and the results are presented in the order asked in the survey. Our initial invitation to participate in the process was sent to all 1094 SBRN members. Of these, 390 SBRN members opened the email (36%), 134 SBRN members (12% of total and 34% of those that opened the email) indicated interest, and 87 (8% of total and 22% of those that opened the email) completed the survey, including Steering Committee members (*n* = 9). Respondents were from 20 countries and represented researchers, trainees, graduate students, practitioners and government employees (see Terminology Consensus Project Participant list below for specific affiliations). Survey results regarding clarity and agreement with the draft definitions, caveats and examples are provided in Table 4. Levels of agreement with the proposed conceptual model and all definitions, caveats and examples were very high (i.e., average ≥ 92%, see Table 4), indicating strong support overall, even before modifications were made to address survey feedback. One survey respondent requested their name be removed as a project participant because of disagreements with the paper content and two others because they felt they had not made a sufficient contribution to warrant coauthorship. In each of these cases the survey results were retained in analyses. In the end, survey results for 87 participants are reported while the manuscript has 84 Terminology Consensus Project coauthors.

**Table 4:** Survey results for clarity and agreement with the draft definitions, caveats and examples

Item	Item clearly stated				Agreement with item			
	Total n	Strongly agree	Somewhat agree	Combined agreement	Total n	Strongly agree	Somewhat agree	Combined agreement
Figure	85	45 (53%)	37 (44%)	82 (96%)	85	41 (48%)	33 (39%)	74 (87%)
Stationary Behavior								
Definition	86	62 (72%)	17 (20%)	79 (92%)	84	56 (67%)	20 (24%)	65 (76%)
Caveats	85	49 (58%)	25 (29%)	74 (87%)	86	51 (59%)	23 (27%)	74 (86%)
Examples	83	52 (63%)	20 (24%)	72 (87%)	84	51 (61%)	29 (35%)	80 (95%)
Sedentary Behavior								
Definition	86	74 (86%)	12 (14%)	86 (100%)	86	74 (86%)	10 (12%)	84 (98%)
Caveats	86	63 (73%)	21 (24%)	84 (98%)	86	68 (79%)	14 (16%)	82 (95%)
Examples	86	72 (84%)	12 (14%)	84 (98%)	86	69 (80%)	15 (17%)	84 (98%)
Standing Still								
Definition	85	64 (75%)	17 (20%)	81 (95%)	86	65 (76%)	19 (22%)	84 (98%)
Caveats	86	69 (80%)	12 (14%)	81 (94%)	86	69 (80%)	12 (14%)	81 (94%)
Examples	85	65 (76%)	18 (21%)	83 (98%)	86	66 (77%)	19 (22%)	85 (99%)
Screen Time								
Definition	85	70 (82%)	12 (14%)	82 (96%)	84	71 (85%)	10 (12%)	81 (96%)
Caveats	84	60 (71%)	17 (20%)	77 (92%)	82	60 (73%)	15 (18%)	75 (91%)
Examples	84	65 (77%)	14 (20%)	79 (94%)	83	62 (75%)	15 (18%)	77 (93%)
Non-Screen-Based Sedentary Time								
Definition	85	73 (86%)	9 (11%)	82 (96%)	85	71 (84%)	8 (9%)	79 (93%)
Caveats	85	66 (78%)	11 (13%)	77 (91%)	86	64 (74%)	15 (17%)	79 (92%)
Examples	85	66 (78%)	13 (15%)	79 (93%)	86	63 (73%)	14 (16%)	77 (90%)
Sitting								
Definition	85	72 (85%)	9 (11%)	81 (95%)	86	70 (81%)	11 (13%)	81 (94%)
Caveats	85	14 (16%)	11 (13%)	75 (88%)	84	59 (70%)	15 (18%)	74 (88%)
Examples	85	61 (72%)	14 (16%)	75 (88%)	85	55 (65%)	21 (25%)	76 (89%)
Reclining								
Definition	84	68 (81%)	10 (12%)	78 (93%)	83	68 (82%)	8 (10%)	76 (92%)
Caveats	84	70 (83%)	8 (10%)	78 (93%)	83	69 (83%)	7 (8%)	76 (92%)
Examples	84	70 (83%)	9 (11%)	79 (94%)	83	67 (81%)	10 (12%)	77 (93%)
Lying								
Definition	85	75 (88%)	6 (7%)	81 (95%)	85	75 (88%)	6 (7%)	81 (95%)
Caveats	84	76 (90%)	3 (4%)	79 (94%)	85	76 (89%)	4 (5%)	80 (94%)
Examples	85	75 (88%)	6 (7%)	81 (95%)	85	75 (88%)	6 (7%)	81 (95%)
Sedentary Behavior Pattern								
Definition	86	78 (91%)	6 (7%)	84 (98%)	86	76 (88%)	9 (10%)	85 (99%)
Caveats	86	72 (84%)	9 (10%)	81 (94%)	85	71 (84%)	9 (11%)	80 (94%)
Examples	86	60 (70%)	19 (22%)	79 (92%)	85	58 (68%)	19 (22%)	77 (91%)
Average								
Definition		71 (83%)	11 (13%)	82 (96%)		70 (82%)	11 (13%)	81 (95%)
Caveats		65 (77%)	13 (15%)	78 (92%)		65 (77%)	13 (15%)	78 (92%)
Examples		65 (77%)	14 (16%)	79 (93%)		63 (74%)	16 (19%)	79 (94%)

Total sample size was 87 – not all respondents answered all survey questions so individual rows have varying “n”.

Other response categories had mostly empty cells as is evident from the very high prevalence of agreement. Complete data are available upon request

**Fig. 2:**
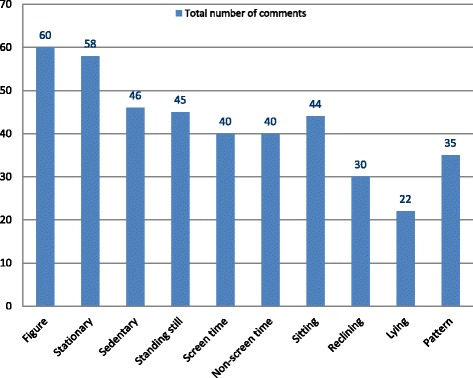
Distribution of respondents’ comments over each survey section (survey 3 from Fig. 1; full survey provided in Additional file 1)

In addition to the closed-ended question results presented in Table 4, survey respondents were given the opportunity to provide written comments on all aspects of the consensus project. A total of 420 comments from the respondents were collected (see Fig. 2 for distribution of written responses). With respect to the conceptual model circulated in the survey (see Additional file 1), 17 respondents expressed concerns about the amount of space allocated to each type of activity (e.g., sleep, physical activity, stationary time); and the use of colors. Six respondents commented that it was unclear what time, bouts, interruptions and/or breaks meant in the figure, and five suggested to move or link *standing still* to the *light physical activity* section. Finally, four respondents had concerns about the title of the figure because it contained “24-h movement behaviors” but was including a lot of “non-movement behaviors”.

Concerning the definition and caveats for *stationary behavior* bouts and breaks, seven respondents asked to specify a minimum and a maximum amount of time for a bout and/or break. Four respondents suggested organizing the examples by presenting the most common types of actions first and two asked to put s*tanding still* in the *light physical activity* category. For the definitions related to *sedentary behavior*, four respondents asked again to specify duration of bout and/or break and three suggested that we should use the interval of 1 to 1.5 METs, instead of ≤1.5 METs. The most recurrent feedback for the *standing still* section concerned the proposed MET value (≤2.0 METs): six respondents thought that it was too high, too low, or not adapted to everyone, and two suggested adding the definition of 1 MET in this section. Two respondents were against the use of the term “standing still” given that one may be moving weight from one leg to the other when standing, and again, two suggested to put this definition in the *light physical activity* section. Similar to the previous sections, two respondents asked for a specific length of time for a bout and/or break. Lastly, two criticized the inclusion of “using a standing desk” as an example of *standing still*.

With regard to the definitions related to *screen time*, there were a few comments about the examples used: six respondents asked for and suggested more examples, and three suggested specifically adding “video games” in the examples. For *non-screen-based sedentary time*, seven respondents also gave example suggestions, and four asked for more consistency in the examples (concerning the wording, the hierarchical organization and the use of the “non-recreational/occupational” language present in other sections).

Several relevant comments were made in the sections concerning the proposed definitions for *sitting*, *reclining* and *lying*. For the *sitting* definition, 15 respondents asked for or suggested further clarifications and examples for active sitting and five for its distinction with passive sitting. Seven respondents also expressed concerns over the evidence supporting the proposed MET-value, and five asked to add specific examples for persons with disabilities, and for infants and toddlers. Concerning the *reclining* definition, four respondents asked for or suggested more examples, three said that reclining does not have to be “relaxed” or passive, and two wanted to state that body weight is supported by some particular parts of the body (e.g., lower back, buttocks). Finally, for *lying*, seven respondents suggested clarifying being asleep or being awake in the caveats, three asked for or suggested examples (especially for children) and two suggested to specify the distinction between active and passive *lying*, as it was done for *sitting*. Finally, concerning the *sedentary behavior pattern* definition and caveats, 21 respondents asked for the definition of specific values, cut-points or details for the definitions of breaker, prolonger, extended sitting bout, the distinction between long- and short-bouts, and frequent breaks.

After analysis of the qualitative and quantitative survey data, feedback from the participating SBRN members was taken into account and several major edits were made to the Figure and the definitions by the Steering Committee. First, the conceptual model colors were reorganized and an explanation was added stating that the proportion of space is not representative of the recommended time that should be spent in each behavior category. The definitions were modified to ensure all examples were organized in a consistent fashion, with the most common examples occurring first; the *standing still* term was changed to *standing* with clarifications of passive standing and active standing (both stationary behaviors); and examples of active screen time were added to the *screen time* definition. Changes to definitions were recirculated to all Terminology Consensus Project participants with the draft manuscript and additional reviews and comments were received. These reviews led to several additional minor changes to definitions, caveats and examples. The standing definition was significantly modified with a clarification and examples of passive vs active standing added. The operational definition of “MET” was also added as a footnote to the Table 5 list of definitions. Finally, the conceptual model was further refined based on comments received, removing the term “stationary behavior” and rearranging the illustration to explicitly present the dual components of energy expenditure (inner ring) and posture (outer ring).

**Table 5:** Final definitions, caveats and examples of key terms from the Sedentary Behavior Research Network (SBRN) Terminology Consensus Project

Term	1. Physical Inactivity
General definition	An insufficient physical activity level to meet present physical activity recommendations [45, 80, 81].
Caveats	General definition applies to all age and ability groups.
Examples	• Toddlers and preschoolers (1–4 years): Not achieving 180 min of physical activity of any intensity per day [45]. • Children and youth (5–17 years): Not achieving 60 min of moderate- to vigorous-intensity physical activity per day [81]. • Adults (≥ 18 years): Not achieving 150 min of moderate-to-vigorous-intensity physical activity per week or 75 min of vigorous-intensity physical activity per week or an equivalent combination of moderate- and vigorous-intensity activity [81].
Term	2. Stationary Behavior
General definition	Stationary behavior refers to any waking behavior done while lying, reclining, sitting, or standing, with no ambulation, irrespective of energy expenditure.
Caveats	• Stationary time: The time spent for any duration (e.g., per day, per week), in any context (e.g., at school/work), and at any intensity (e.g., standing in a line, working on an assembly line with no ambulation, working at a standing desk, sitting in a classroom) in stationary behaviors. • Stationary bout: A period of uninterrupted stationary time. • Stationary interruptions/breaks: A non-stationary bout in between two stationary bouts (applies to all age and ability groups except infants). General definition applies to all age and ability groups except for infants (<1 year to pre-walking) and people with a mobility impairment who are unable to stand.
Examples	**•** Use of electronic devices (e.g., television, computer, tablet, phone) while sitting, reclining or lying; reading/writing/drawing/painting/talking while sitting; sitting at school/work; sitting in a bus, car or train. **•** Standing in a line; standing at church; standing for a hallway discussion; writing a text-message while standing; using a standing desk. **•** Being carried/held/cuddled by someone.
Term	3. Sedentary Behavior
General definition	Sedentary behavior is any waking behavior characterized by an energy expenditure ≤1.5 metabolic equivalents (METs), while in a sitting, reclining or lying posture [25].
Caveats	• Sedentary time: The time spent for any duration (e.g., minutes per day) or in any context (e.g., at school or work) in sedentary behaviors. • Sedentary bout: A period of uninterrupted sedentary time [17, 37]. • Sedentary interruptions/breaks: A non-sedentary bout in between two sedentary bouts. • Infants (<1 year or pre-walking): Any waking behavior characterized by low energy expenditure while restrained (e.g., stroller/pram, high chair, car seat/capsule), or when sedate (e.g., reclining/sitting in a chair with little movement but not restrained). Time spent in the prone position (“tummy time”) is not considered a sedentary exposure. • Toddlers [51] and preschoolers (1–4 years), children and youth (5–17 years) [48–52] adults (≥ 18 years) and all ability groups [82]: Same as the general definition.
Examples	• Infants (<1 year or pre-walking): Lying awake in the bed with minimal movement; sitting in a baby chair/high chair/stroller/car seat with minimal movement; being carried/held/cuddled by someone • Toddlers and preschoolers (1–4 years): Use of electronic devices (e.g., television, computer, tablet, phone) while sitting, reclining or lying; reading/drawing/painting while sitting; sitting in stroller; sitting in baby chair or couch while eating a meal; sitting in a bus, car or train. • Children and youth (5–17 years): Use of electronic devices (e.g., television, computer, tablet, phone) while sitting, reclining or lying; reading/writing/drawing/painting while sitting; homework while sitting; sitting at school; sitting in a bus, car or train. • Adults (≥ 18 years): Use of electronic devices (e.g., television, computer, tablet, phone) while sitting, reclining or lying; reading/writing/talking while sitting; sitting in a bus, car or train. • People who use a manual wheelchair or a power chair: Use of electronic devices (e.g., television, computer, tablet, phone) while sitting, reclining or lying; reading/writing/drawing/painting/talking while sitting; sitting in a bus, car or train; moving from place to place in a power chair; being pushed while passively sitting in a manual wheelchair.
Term	4. Standing
General definition	A position in which one has or is maintaining an upright position while supported by one’s feet [83].
Caveats	• Active standing: Active standing refers to any waking activity in a standing posture characterized by an energy expenditure >2.0 METs, while standing without ambulation, whether supported or unsupported. • Passive standing: Passive standing refers to any waking activity in a standing posture characterized by an energy expenditure ≤2.0 METs, while standing without ambulation, whether supported or unsupported [84]. • Standing time: The time spent for any duration (e.g., minutes per day) or in any context (e.g., at school/work) while standing. • Standing bout: A period of uninterrupted time while standing. • Standing interruptions/breaks: A non-standing bout in between two standing bouts. • Infants (<1 year or pre-walking), toddlers and preschoolers (1–4 years), children and youth (5–17 years), adults (≥ 18 years) and people who use a manual wheelchair or a power chair: Same as the general definition. • People who are unable to stand: Not applicable.
Examples	• Active standing: Standing on a ladder; standing while painting; standing while washing dishes; working an assembly line while standing; standing while juggling; standing while lifting weights. • Passive standing: Standing in a line; standing for a hallway discussion; use of electronic devices (e.g., television, computer, tablet, phone) while standing; standing at church. • Supported standing: Standing while holding a couch, chair, or a parent’s hand; standing with the aid of crutches, a cane, standing frame or body weight support.
Term	5. Screen Time
General definition	Screen time refers to the time spent on screen-based behaviors [15, 85]. These behaviors can be performed while being sedentary or physically active.
Caveats	• Recreational screen time: Time spent in screen behaviors that are not related to school or work [44]. • Stationary screen time: Time spent using a screen-based device (e.g., smartphone, tablet, computer, television) while being stationary in any context (e.g., school, work, recreational). • Sedentary screen time: Time spent using a screen-based device (e.g., smartphone, tablet, computer, television) while being sedentary in any context (e.g., school, work, recreational). • Active screen time: Time spent using a screen-based device (e.g., smartphone, tablet, computer, television) while not being stationary in any context (e.g., school, work, recreational). • General definition applies to all age and ability groups.
Examples	• All age and ability groups: Watching TV, using a smartphone/tablet, using a computer. • Active screen time: Playing active video games, running on a treadmill while watching television.
Term	6. Non-Screen-Based Sedentary Time
General definition	Non-screen-based sedentary time refers to the time spent in sedentary behaviors that do not involve the use of screens.
Caveats	• Recreational non-screen time: Time spent in non-screen based sedentary behaviors that are not related to school or work. • General definition applies to all age and ability groups.
Examples	• Infants (<1 year or pre-walking): Lying supine on a mat while sedate; sitting in a stroller or car seat with little movement. • Toddlers and preschoolers (1–4 years): Sitting in a child seat, chair or car seat; sitting idle in the sandbox or on the floor; reading a non-electronic book or playing a board game while seated. • Children and youth (5–17 years): Sitting at school; sitting doing homework or art work; reading a non-electronic book; playing a board game; sitting in a car. • Adults (≥ 18 years): Reading a non-electronic book; playing a board game; sitting in a car. • People who use a manual wheelchair or a power chair: Reading a non-electronic book; playing a board game; sitting in a car; being pushed while passively sitting in a manual wheelchair.
Term	7. Sitting
General definition	A position in which one’s weight is supported by one’s buttocks rather than one’s feet, and in which one’s back is upright [83].
Caveats	• Active sitting: Active sitting refers to any waking activity in a sitting posture characterized by an energy expenditure >1.5 METs. • Passive sitting: Passive sitting refers to any waking activity in a sitting posture characterized by an energy expenditure ≤1.5 METs. • General definition applies to all age and ability groups.
Examples	• Active sitting: Working on a seated assembly line; playing guitar while seated; using devices that engage ones feet/legs while seated; doing arm ergometry while in a wheelchair. • Passive sitting: Refer to sedentary behavior examples while sitting.
Term	8. Reclining
General definition	Reclining is a body position between sitting and lying.
Caveats	General definition applies to all age and ability groups. Reclining behavior can be either passive (≤ 1.5 METs) or active (>1.5 METs).
Examples	Passive reclining (all age and ability groups): Lounging/slouching on a chair or couch while sedentary. Active reclining (all age and ability groups): Recumbent cycling.
Term	8. Lying
General definition	Lying refers to being in a horizontal position on a supporting surface [83].
Caveats	General definition applies to all age and ability groups. Lying behavior can be either passive (≤ 1.5 METs) or active (>1.5 METs).
Examples	Passive lying (all age and ability groups): Lying on a couch, bed or floor while sedentary. Active lying (all age and ability groups): Isometric plank hold.
Term	9. Sedentary Behavior Pattern
General definition	The manner in which sedentary behavior is accumulated throughout the day or week while awake (e.g., the timing, duration and frequency of sedentary bouts and breaks) [19, 69].
Caveats	General definition applies to all age and ability groups.
Examples	Prolonger: Someone who accumulates sedentary time in extended continuous bouts [1]. Breaker: Someone who accumulates sedentary time with frequent interruptions and in short bouts [1].

MET = metabolic equivalent corresponding to resting metabolic rate of the population under study. A metabolic equivalent is deemed to be 3.5 ml O_2_/kg/min in adults without mobility impairment or chronic disease. A metabolic equivalent is generally higher in children and in those with conditions that elevate muscle activity or metabolism and is generally lower in those with paralysis, small muscle mass or wasting conditions. The interpretation of MET values should be made with attention to the population under study, and the definitions and caveats above applied accordingly

The further revised manuscript, table of definitions, and conceptual model was recirculated to all participants for a final review and sign-off. In conjunction with this final review, participants were also asked three questions: 1. Do you agree that your view has been listened to and taken account during this process? (yes/no) 2. Has your level of agreement/support for the consensus definitions changed since you completed the survey? (increased/decreased/no change) 3. Are you likely to use the consensus definitions? (yes/no). Of the 84 final coauthors 100% answered “yes” to questions one and three providing strong support for the participatory consensus process. For question two, 73% responded “increased” and 27% responded “no change”, no one responded decreased. With the already strong support reported in response to the initial survey it is reasonable to believe that the revisions made in response to feedback received resulted in final definitions and a conceptual model that has very strong support from participants. Subjective comments received with participant responses to the various reviews support this observation.

#### Final conceptual model and consensus definitions

**Fig. 3:**
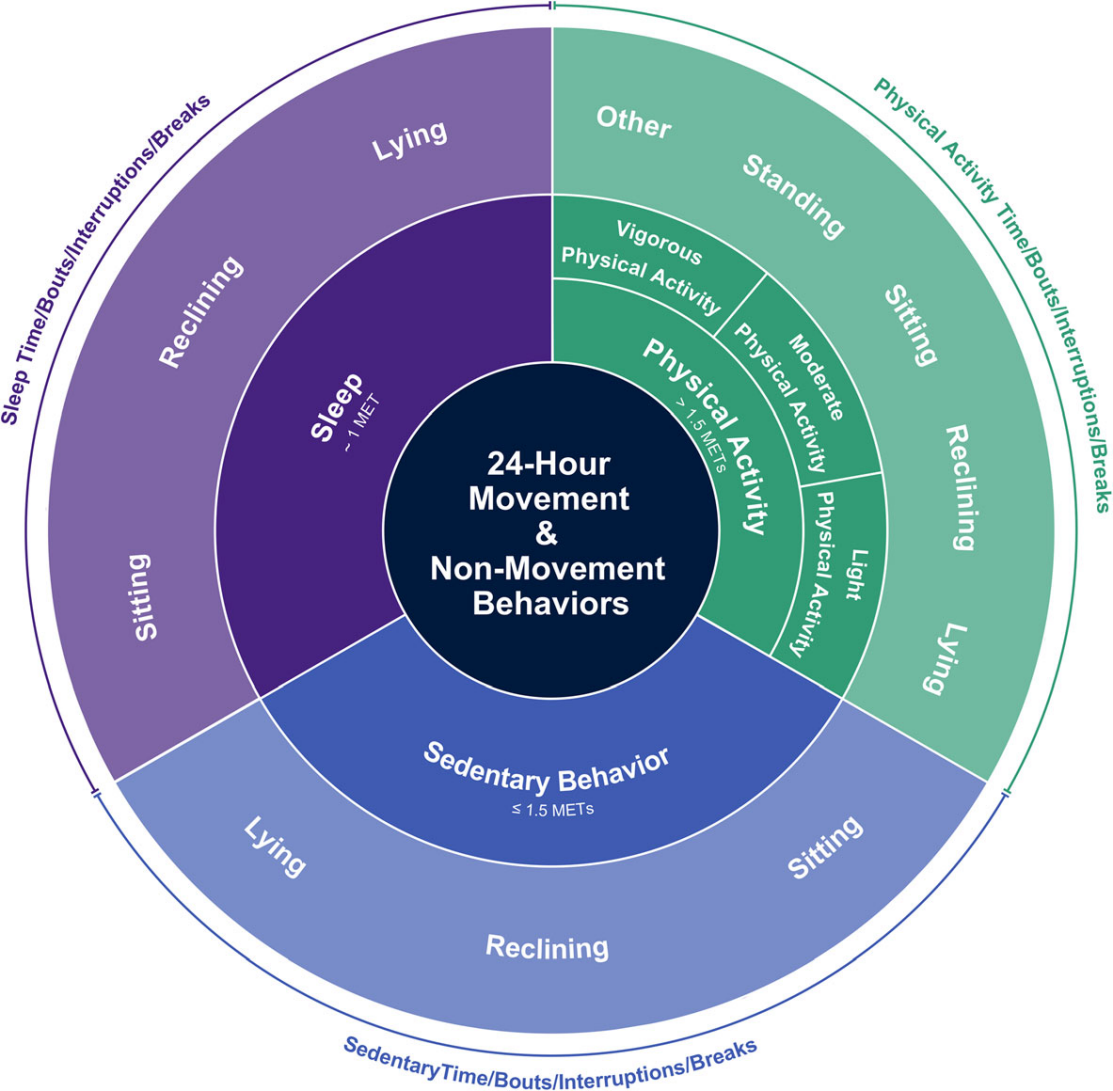
Illustration of the final conceptual model of movement-based terminology arranged around a 24-h period. The figure organizes the movements that take place throughout the day into two components: The inner ring represents the main behavior categories using energy expenditure. The outer ring provides general categories using posture. Detailed definitions, caveats and examples related to sedentary behavior are provided in Table 5. The proportion of space occupied by each behavior in this figure is not prescriptive of the time that should be spent in these behaviors each day

The final conceptual model, presented in Fig. 3, illustrates how the various terms fit together structurally and provides support for the definitions included in this project. Final consensus definitions for key terms (physical inactivity, stationary behavior, sedentary behavior, standing, screen time, non-screen-based sedentary time, sitting, reclining, lying, sedentary behavior pattern), caveats related to age and/or ability differences, examples and related references are provided in Table 5. For the purpose of these consensus definitions, the following age groups were used: Infants – ages 3 months to <1 year; Toddlers and preschoolers – ages 1 to 4 years; Children and youth – ages 5 to 17 years; Adults and Older Adults – age ≥ 18 years. The age ranges for 1- to 4-year-olds and for children and youth are consistent with those used in the Canadian Physical Activity Guidelines for the Early Years and the Canadian 24-Hour Movement Guidelines for Children and Youth respectively [44, 45]. Adults and Older Adults were grouped given that definitions did not differ across the adult lifespan. The definitions were developed with attention to being inclusive in language and examples. Specifically, examples of interpretations of definitions for people with mobility impairment(s) are provided where appropriate.

## Discussion

In this paper we describe the processes and outcomes of a comprehensive initiative to establish consensus definitions for terms relevant to sedentary behavior research and to develop a conceptual model to illustrate the hierarchical structural connections among the various terms. This Terminology Consensus Project had several novel and progressive elements: it was informed by the best available evidence; it built on the previous SBRN definition project [25] and other related initiatives (Table 2); it leveraged the diverse membership base of SBRN using an open participatory process; it significantly expanded the scope of terminology covered compared to earlier efforts; it developed a conceptual model to illustrate the structural connections among the various terms; it was observant of nuances for all age groups; it added examples to help interpret the terms; and it was attentive to the interpretation and application of the terminology for people with varying physical abilities. SBRN now has 1273 members from 35 countries (as of March 20, 2017) representing all inhabited continents, of which 84 members from 20 countries and multiple employment sectors participated in all aspects of this project (see Terminology Consensus Participants section below for details).

This project resulted in several notable advances in terminology standardization in the field of sedentary behavior. For example, the addition of the term “lying” to the widely used definition of sedentary behavior [25] fills an obvious gap in the earlier definition. The introduction of the term “stationary behavior” provides a behavioral classification home for “standing”. The consensus definition suggests “passive standing” is a stationary, non-sedentary behavior ≤2.0 METs and that “active standing” is a stationary physical activity >2.0 METs (see Table 5 definitions for more details). Also, for clarity, researchers can now use the term “stationary time” when reporting data collected from an accelerometer that does not measure posture. The consensus definitions add clarity on the distinction between sedentary “behaviors” (with context) and “time” at an established level of intensity (absent of context, which includes most accelerometer and inclinometer data) as well as a standardized approach for defining bouts and interruptions/breaks. Although there is currently no consensus on a minimum duration of a sedentary bout, the work of Kim et al. [37] provide support for 10 min as a conservative sedentary bout threshold. The applicability of these movement behaviors for individuals with mobility impairment was also a distinct improvement.

The conceptual model, with clear definitions of terminology related to sedentary behavior, provides clarity for researchers interested in exploring the relationships between, and among, the various movement behaviors across the whole day (i.e., sleep, sedentary behaviors and various intensities of physical activity) [44, 46] and may facilitate future research exploring behavior substitutions aimed to improve health [47]. Consensus definitions, as presented here, were derived to assist with the standardization, or at least harmonization, of measurement procedures, data processing, and data analytics. Ultimately, common definitions form the foundation upon which related measurement decisions are based. It is hoped that these consensus definitions will help facilitate standardization efforts, reduce confusion, and advance initiatives and research related to sedentary behavior.

There has been much debate around the appropriate MET threshold to use when describing or characterizing sedentary behavior and standing. While there will always be exceptions, the consensus definitions proposed in this paper (sedentary behavior ≤1.5 METs, passive standing ≤2.0 METs; Table 5) have a broad base of support. Several recent studies have shown that a variety of sedentary behaviors were ≤1.5 METs for children and youth [21, 48–52], while others suggested that the threshold may be too low for many childhood behaviors that would be considered sedentary by observation [53, 54]. There is general agreement that determining METs in children should use a VO_2_ standard that is higher than 3.5 ml/kg/min [55], and when this is employed, there is broad support for the ≤1.5 MET threshold for preschool-aged [21, 51] and school-aged children and youth [48–50, 52]. For adults, the sedentary behavior threshold of ≤1.5 METs has been widely recommended and accepted [4, 7, 25, 56].

The MET threshold for passive standing (e.g., not working on an assembly line or painting) is also supported by the literature. For example, the compendium of energy expenditures for youth reports the following MET values: sending text messages while standing, 1.8 METs; talking while standing, 1.8 METs; standing quietly, 1.5 METs; arts and crafts while standing, 1.9 METs; reading while standing, 1.8 METs; drinking while standing, 2.0 METs; and eating while standing, 2.0 METs [55]. The standing threshold of ≤2.0 METs for adults also has support in the literature [29, 31]. Values from the highly cited physical activity compendium also support this threshold, for example: standing doing miscellaneous, 2.0 METs; standing doing arts and crafts (light effort), 1.8 METs; cooking or food preparation while standing, 2.0 METs; standing quietly in a line, 1.2 METs; standing while talking or talking on the phone, 1.8 METs; and standing while reading, 1.8 METs [57].

### Strengths and limitations

A strength of this project was the systematic approach taken to determine the list of key terms and arrive at consensus definitions for these terms. The use of a large, broad, diverse and global cohort of participants to provide input to the consensus definitions is a clear strength and should help with the uptake and acceptance of these definitions. Yet, the participants were limited to SBRN members and the overall participation rate by SBRN members was only 8%. However, for SBRN members that opened the email invitation to participate, the rate was 22%, slightly lower than the participation rate of 34% (52/155) for the first SBRN consensus definitions project [25]. It is possible that the participants do not represent the larger population of sedentary behavior researchers and practitioners. A limitation to work of this nature is that there will always be situations and/or circumstances where the proposed definitions are sub-optimal or not wholly satisfactory. Furthermore, there will likely be different conceptual models and other definitions that exist for good reasons or that will emerge as research evolves. The goal of this project was not to marginalize such opinions, but rather respond to calls for better standardization and harmonization of work in the field at this point in time.

### Future directions and research needs

There are many future research needs directly related to the definitions proposed in this manuscript. For example, the validity of the proposed terms needs to be tested and further work is necessary to study their content and face validity. In addition, refinements of MET-value thresholds for different ages (e.g., young adults vs older adults), different situations (e.g., while sitting and typing; in different environmental conditions; or in short vs extended durations) or as measured using alternate measurement methods (e.g., measuring muscle activity), and the consequent MET-value-related health implications, requires further exploration [29, 32]. The importance of sedentary behavior context (e.g., leisure time vs school vs work) and modality (e.g., television viewing vs reading vs talking on the phone) in relation to health indicators and outcomes is poorly understood and additional clarity may lead to refinement of the conceptual model and/or the consensus definitions. Additional research is required in populations with mobility impairments or disease conditions where different cut-points/thresholds may exist and there may be different relationships with health indicators than in the general population. Research into the health impact of standing is just beginning to emerge [58–63]. How standing interacts with sedentary behaviors and/or sedentary time, sleep and physical activities of various intensities, and how these interactions relate to health outcomes and indicators, needs further research. Through the survey responses SBRN members asked for more research to arrive at specific MET-values and related accelerometer cut-points for categorizing sedentary time. Further research to understand and compare accelerometer data collected from different placement locations (e.g., hip vs wrist) when assessing sedentary time is also required. There were also requests from respondents for standard criteria for defining sedentary bouts and breaks to classify people as “Breakers” or “Prolongers” [1]. Similarly, further research exploring health indicators and outcomes associated with sitting bouts of different durations, distinguishing between long- and short-bouts, and operationalizing “frequent breaks” were all recommended. How the intensity of movement of a “break” affects resultant health-related indictors also requires further exploration. Although questionnaires are frequently used to assess sedentary behavior, few have undergone psychometric testing [10]. Further research is needed to identify the most valid and reliable means of assessing total sedentary behaviour, specific forms of sedentary behaviour, and sedentary behaviour patterns via self- and proxy-report questionnaires. Finally, the physiological impact of ‘transitions’ (e.g., sleep-awake, sit-stand, stand-step) needs to be elucidated [64].

## Conclusion

The definitions arrived at through the SBRN Terminology Consensus Project are presented as standardized definitions that we encourage researchers to embrace, use and promote. With the assistance of international SBRN volunteers, efforts are underway to ensure these definitions are translated into several other languages, including French, Spanish, Portuguese, Dutch, Korean, German and others. Copies of all language versions of the consensus definitions are available on the SBRN website [24]. Periodic reviews of these consensus definitions should be done, and updates made when appropriate. It is hoped that the definitions resulting from this comprehensive, transparent and broad-based participatory process will result in widely supported and adopted standardization of terminology, and advance future research, interventions, policies and practices related to sedentary behaviors.

## Additional file

Additional file 1: SBRN Terminology Consensus Project survey. (DOCX 435 kb)
