# Supplementary material for: Sedentary Behavior Research Network (SBRN) – Terminology Consensus Project process and outcome
Source: Int J Behav Nutr Phys Act. 2017 Jun 10;14:75. doi: 10.1186/s12966-017-0525-8 (PMC5466781; doi:10.1186/s12966-017-0525-8)
Supplement: Additional file 1: — SBRN Terminology Consensus Project survey. (DOCX 435 kb) [file 12966_2017_525_MOESM1_ESM.docx]

**Appendix A:** SBRN Terminology Consensus Project survey.

Updated SBRN Definition Consensus Statement

You are being invited to participate in a survey soliciting your input on an updated **SBRN Definition Consensus Statement**. You are being invited to participate because you responded with interest in being involved in this project when asked in the SBRN email back in April 2016. 

This stakeholder survey will be open until **September 30th, 2016 at 17:00 EST**. The survey should take you no longer than 20 minutes to complete. On the next series of screens you will be presented first with a conceptual illustration followed by nine proposed definitions for the updated SBRN Definition Consensus Statement (along with caveats and examples) – there are 10 sets of screens in total. You may advance or go back using the buttons on the bottom of the screen.

Participation in this survey is voluntary. By accessing and completing this survey you are giving your implied/passive consent to participate in the survey. Supportive comments and substantiated suggestions are welcomed. The updated SBRN Definition Consensus Statement along with a summary of the findings from this survey will be published in a peer-reviewed journal. If you complete this survey and identify yourself at the end of the survey, you will be listed as one of the authors on the peer-reviewed paper (which you will be sent for review and feedback before submission).

If you have any questions about this study, please contact Dr. Mark Tremblay at 613-737-7600 ext 4114 or [mtremblay@cheo.on.ca](mailto:mtremblay@cheo.on.ca). 

**Note: This updated SBRN Definition Consensus Statement is in draft form and not intended for general circulation.**

| Figure | 1. Diagram Integrating Related Terms in the Context of the [Canadian 24-Hour Movement Guidelines for Children and Youth](http://www.csep.ca/en/guidelines/24-hour-movement-guidelines)^1^, with Focus on Sedentary-Related Terms |
| --- | --- |
| Illustration | 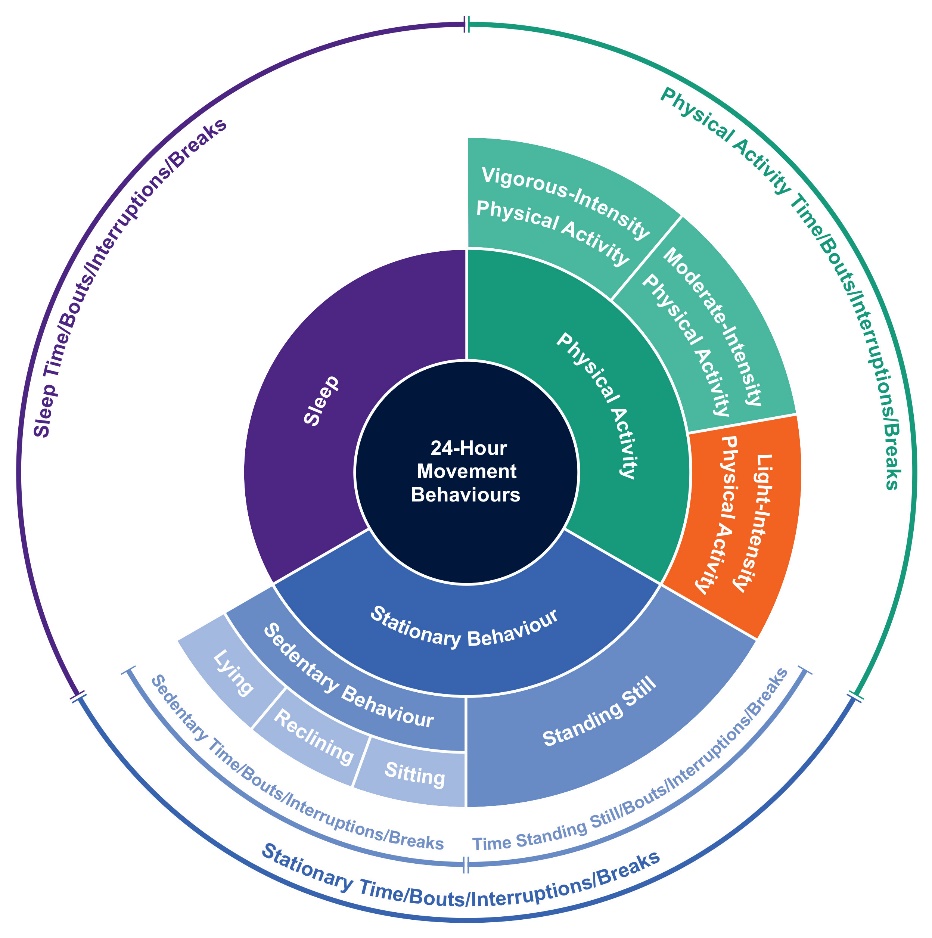 |
| References | ^1^ Tremblay MS, Carson V, Chaput JP, Connor Gorber S, Dinh T, Duggan M, Faulkner G, Gray CE, Gruber R, Janson K, Janssen I, Katzmarzyk PT, Kho ME, Latimer-Cheung AE, LeBlanc C, Okely AD, Olds T, Pate RR, Phillips A, Poitras VJ, Rodenburg S, Sampson M, Saunders TJ, Stone JA, Stratton G, Weiss SK, Zehr L. Canadian 24-Hour Movement Guidelines for Children and Youth: An Integration of Physical Activity, Sedentary Behaviour, and Sleep. Appl Physiol Nutr Metab. 2016 Jun;41(6 Suppl 3):S311-27. |

**Is the figure clearly stated?**

|  | Strongly agree | Somewhat agree | Neither agree or disagree | Somewhat disagree | Strongly disagree |
| --- | --- | --- | --- | --- | --- |
|  |  |  |  |  |  |

**Do you agree with the figure?**

|  | Strongly agree | Somewhat agree | Neither agree or disagree | Somewhat disagree | Strongly disagree |
| --- | --- | --- | --- | --- | --- |
|  |  |  |  |  |  |

**In the box below, please enter any comments that you would like to add regarding the figure.**

| **Term** | 1. Stationary Behaviour |
| --- | --- |
| **General Definition** | Stationary behaviour refers to any behaviour done while being sedentary or standing still. |
| **Caveats** | - **Stationary time:** The time spent for any duration (e.g., per day, per week) or in any context (e.g., at school/work) in stationary behaviours. - **Stationary bout:** A period of uninterrupted stationary time. - **Stationary interruptions/breaks:** A non-stationary bout in between two stationary bouts (applies to all age and ability groups except infants). - General definition applies to all age and ability groups except for infants (<1 year to pre-walking), and people with a mobility impairment who are unable to stand. - For someone with a mobility impairment or condition for whom standing is effortful (e.g., >2 METs), this behaviour would not be considered a stationary behaviour. |
| **Examples** | - Being carried/held/cuddled by someone. - Standing in a line; standing at church; standing for a hallway discussion; writing a text-message while standing still; using a standing desk. - Use of electronic devices (e.g., television, computer, tablet, phone) while sitting, reclining or lying; reading/writing/drawing/painting/talking while sitting; sitting at school/work; sitting in a bus, car or train. |
| **References** |  |

**Are the following items clearly stated?**

|  | Strongly agree | Somewhat agree | Neither agree or disagree | Somewhat disagree | Strongly disagree |
| --- | --- | --- | --- | --- | --- |
| General Definition |  |  |  |  |  |
| Caveats |  |  |  |  |  |
| Examples |  |  |  |  |  |

**Do you agree with the following items?**

|  | Strongly agree | Somewhat agree | Neither agree or disagree | Somewhat disagree | Strongly disagree |
| --- | --- | --- | --- | --- | --- |
| General Definition |  |  |  |  |  |
| Caveats |  |  |  |  |  |
| Examples |  |  |  |  |  |

**In the box below, please enter any comments that you would like to add regarding this definition.**

| **Term** | 1. Sedentary Behaviour |
| --- | --- |
| **General Definition** | Sedentary behaviour is any waking behaviour characterized by an energy expenditure ≤1.5 metabolic equivalents (METs), while in a sitting, reclining or lying posture.^1^ |
| **Caveats** | - **Sedentary time:** The time spent for any duration (e.g., minutes per day) or in any context (e.g., at school) in sedentary behaviours. - **Sedentary bout:** A period of uninterrupted sedentary time.^2^ - **Sedentary interruptions/breaks:** A non-sedentary bout in between two sedentary bouts. - **Infants (<1 year or pre-walking):** Any waking behaviour characterized by low energy expenditure while restrained (e.g., stroller/pram, high chair, car seat/capsule), or when sedate (e.g., reclining/sitting in a chair with little movement but not restrained).  Time spent in the prone position (“tummy time”) is not considered a sedentary exposure. - **Toddlers^3^ and preschoolers (1-4 years), children and youth (5-17 years)^3-7^, adults (≥ 18 years) and all ability groups:** Same as the general definition. |
| **Examples** | - **Infants (<1 year or pre-walking):** Lying awake in the bed with minimal movement; sitting in a baby chair/high chair/stroller/car seat; being carried/held/cuddled by someone - **Toddlers and preschoolers (1-4 years):** Use of electronic devices (e.g., television, computer, tablet, phone) while sitting, reclining or lying; reading/drawing/painting while sitting; sitting in stroller; sitting in baby chair or couch while eating a meal; sitting in a bus, car or train. - **Children and youth (5-17 years):** Use of electronic devices (e.g., television, computer, tablet, phone) while sitting, reclining or lying; reading/writing/drawing/painting while sitting; homework while sitting; sitting at school; sitting in a bus, car or train. - **Adults (≥ 18 years):**Use of electronic devices (e.g., television, computer, tablet, phone) while sitting, reclining or lying; reading/writing/talking while sitting; sitting in a bus, car or train. - **People who use a manual wheelchair or a power chair:** Use of electronic devices (e.g., television, computer, tablet, phone) while sitting, reclining or lying; reading/writing/drawing/painting/talking while sitting; sitting in a bus, car or train; moving from place to place in a power chair; being pushed while passively sitting in a manual wheelchair. |
| **References** | ^1^ Sedentary Behaviour Research Network. Letter to the editor: standardized use of the terms "sedentary" and "sedentary behaviours". Appl Physiol Nutr Metab. 2012;37(3):540-2. ^2^ Altenburg TM, Chinapaw MJ. Bouts and breaks in children's sedentary time: currently used operational definitions and recommendations for future research. Prev Med. 2015;77:1-3. ^3^ Puyau MR, Adolph AL, Liu Y, Wilson TA, Zakeri IF, Butte NF. Energy cost of activities in preschool-aged children. J Phys Act Health. 2016;13(6 Supplement 1):S11. ^4^ Lau M, Wang L, Acra S, Buchowski MS. Energy expenditure of common sedentary activities in youth." J Phys Act Health. 2016;13(6 Supplement 1):S17. ^5^ Schuna Jr JM, BarreriaTV, Hsia DS, Johnson WD, Tudor-Locke C. Youth energy expenditure during common free-living activities and treadmill walking. J Phys Act Health. 2016;13(6 Supplement 1):S29. ^6^ Gao Y, Sun H, Zhuang J, Zhang J, Ransdell L, Zhu Z, Wang S. Metabolic equivalents of selected sedentary and physical activities in Chinese youth. J Phys Act Health. 2016;13(6 Supplement 1):S48. ^7^ Innerd A, Azevedo LB. The energy expenditure of free-living physical activities in primary schoolchildren. J Phys Act Health. 2016;13(6 Supplement 1):S59. |

**Are the following items clearly stated?**

|  | Strongly agree | Somewhat agree | Neither agree or disagree | Somewhat disagree | Strongly disagree |
| --- | --- | --- | --- | --- | --- |
| General Definition |  |  |  |  |  |
| Caveats |  |  |  |  |  |
| Examples |  |  |  |  |  |

**Do you agree with the following items?**

|  | Strongly agree | Somewhat agree | Neither agree or disagree | Somewhat disagree | Strongly disagree |
| --- | --- | --- | --- | --- | --- |
| General Definition |  |  |  |  |  |
| Caveats |  |  |  |  |  |
| Examples |  |  |  |  |  |

**In the box below, please enter any comments that you would like to add regarding this definition.**

| **Term** | 1. Standing Still |
| --- | --- |
| **General Definition** | Standing still refers to any waking behaviour characterized by an energy expenditure ≤2.0 METs, while standing without ambulation, whether supported or unsupported.^1^ |
| **Caveats** | - **Time standing still:** The time spent for any duration (e.g., minutes per day) or in any context (e.g., at school/work) while standing still. - **Standing still bout:** A period of uninterrupted time while standing still. - **Standing still interruptions/breaks:** A non-standing still bout in between two standing still bouts. - **Infants (<1 year or pre-walking), toddlers and preschoolers (1-4 years), children and youth (5-17 years), adults (≥ 18 years) and people who use a manual wheelchair or a power chair:** Same as the general definition. - **People who are unable to stand:**Not applicable. |
| **Examples** | - Standing in a line; standing at church; standing for a hallway discussion; writing a text-message while standing still; using a standing desk. - Use of electronic devices (e.g., television, computer, tablet, phone) while standing still. - Supported standing: standing while holding a couch, chair, or a parent's hand; standing with the aid of crutches, a cane, standing frame or body weight support. |
| **References** | ^1^ Tudor-Locke C, Schuna JM Jr, Frensham LJ, Proenca M. Changing the way we work: elevating energy expenditure with workstation alternatives. Int J Obes (Lond). 2014 Jun;38(6):755-65. |

**Are the following items clearly stated?**

|  | Strongly agree | Somewhat agree | Neither agree or disagree | Somewhat disagree | Strongly disagree |
| --- | --- | --- | --- | --- | --- |
| General Definition |  |  |  |  |  |
| Caveats |  |  |  |  |  |
| Examples |  |  |  |  |  |

**Do you agree with the following items?**

|  | Strongly agree | Somewhat agree | Neither agree or disagree | Somewhat disagree | Strongly disagree |
| --- | --- | --- | --- | --- | --- |
| General Definition |  |  |  |  |  |
| Caveats |  |  |  |  |  |
| Examples |  |  |  |  |  |

**In the box below, please enter any comments that you would like to add regarding this definition.**

| **Term** | 1. Screen Time |
| --- | --- |
| **General Definition** | Screen time refers to the time spent on screen-based behaviours.^1-2^ These behaviours can be performed while being sedentary or physically active. |
| **Caveats** | - **Recreational screen time:**Time spent in screen behaviours that are not related to school or work.^3^ - **Sedentary screen time:** Time spent using a screen-based device (e.g., smartphone, tablet, computer, television) while being sedentary. - **Stationary screen time:** Time spent using a screen-based device (e.g., smartphone, tablet, computer, television) while stationary. - **Active screen time:** Time spent using a screen-based device (e.g., smartphone, tablet, computer, television) while not stationary. - General definition applies to all age and ability groups. |
| **Examples** | - **All age and ability groups:** Watching TV, using a smartphone/tablet, using a computer. |
| **References** | ^1^ Anderson SE, Economos CD, Must A. Active play and screen time in US children aged 4 to 11 years in relation to sociodemographic and weight status characteristics: a nationally representative cross-sectional analysis. BMC Public Health. 2008 Oct 22;8:366. ^2^ Stamatakis E, Hamer M, Dunstan DW. Screen-based entertainment time, all-cause mortality, and cardiovascular events: population-based study with ongoing mortality and hospital events follow-up. J Am Coll Cardiol. 2011 Jan 18;57(3):292-9. ^3^ Tremblay MS, Carson V, Chaput JP, Connor Gorber S, Dinh T, Duggan M, Faulkner G, Gray CE, Gruber R, Janson K, Janssen I, Katzmarzyk PT, Kho ME, Latimer-Cheung AE, LeBlanc C, Okely AD, Olds T, Pate RR, Phillips A, Poitras VJ, Rodenburg S, Sampson M, Saunders TJ, Stone JA, Stratton G, Weiss SK, Zehr L. Canadian 24-Hour Movement Guidelines for Children and Youth: An Integration of Physical Activity, Sedentary Behaviour, and Sleep. Appl Physiol Nutr Metab. 2016 Jun;41(6 Suppl 3):S311-27. |

**Are the following items clearly stated?**

|  | Strongly agree | Somewhat agree | Neither agree or disagree | Somewhat disagree | Strongly disagree |
| --- | --- | --- | --- | --- | --- |
| General Definition |  |  |  |  |  |
| Caveats |  |  |  |  |  |
| Examples |  |  |  |  |  |

**Do you agree with the following items?**

|  | Strongly agree | Somewhat agree | Neither agree or disagree | Somewhat disagree | Strongly disagree |
| --- | --- | --- | --- | --- | --- |
| General Definition |  |  |  |  |  |
| Caveats |  |  |  |  |  |
| Examples |  |  |  |  |  |

**In the box below, please enter any comments that you would like to add regarding this definition.**

| **Term** | 1. Non-Screen-Based Sedentary Time |
| --- | --- |
| **General Definition** | Non-screen-based sedentary time refers to the time spent in sedentary behaviours that do not involve the use of screens. |
| **Caveats** | - **Recreational non-screen time:**Time spent in non-screen based sedentary behaviours that are not related to school or work. - General definition applies to all age and ability groups. |
| **Examples** | - **Infants (<1 year or pre-walking):** Lying supine on a mat; sitting in a stroller or car seat. - **Toddlers and preschoolers (1-4 years):** Sitting in a child seat, chair or car seat; sitting idle in the sandbox or on the floor; reading a hardcopy book or playing a board game while seated. - **Children and youth (5-17 years):** Sitting at school; sitting doing homework or art work; reading a hardcopy book; playing a board game; sitting in a car. - **Adults (≥ 18 years):**Reading a hardcopy book; playing a board game; sitting in a car. - **People who use a manual wheelchair or a power chair:** Reading a hardcopy book; playing a board game; sitting in a car; being pushed while passively sitting in a manual wheelchair. |
| **References** |  |

**Are the following items clearly stated?**

|  | Strongly agree | Somewhat agree | Neither agree or disagree | Somewhat disagree | Strongly disagree |
| --- | --- | --- | --- | --- | --- |
| General Definition |  |  |  |  |  |
| Caveats |  |  |  |  |  |
| Examples |  |  |  |  |  |

**Do you agree with the following items?**

|  | Strongly agree | Somewhat agree | Neither agree or disagree | Somewhat disagree | Strongly disagree |
| --- | --- | --- | --- | --- | --- |
| General Definition |  |  |  |  |  |
| Caveats |  |  |  |  |  |
| Examples |  |  |  |  |  |

**In the box below, please enter any comments that you would like to add regarding this definition.**

| **Term** | 1. Sitting |
| --- | --- |
| **General Definition** | A position in which one's weight is supported by one's buttocks rather than one's feet, and in which one's back is upright.^1^ |
| **Caveats** | - **Active vs. passive sitting:**Active sitting refers to any waking activity in a sitting posture characterized by an energy expenditure > 1.5 metabolic equivalents (METs). Passive sitting refers to any waking activity in a sitting posture characterized by an energy expenditure ≤ 1.5 METs. - General definition applies to all age and ability groups. |
| **Examples** | - **Active sitting:** Working on a seated assembly line; playing guitar while seated. - **Passive sitting:** Refer to sedentary behaviour examples while sitting. |
| **References** | ^1^ Concise Oxford English Dictionary. |

**Are the following items clearly stated?**

|  | Strongly agree | Somewhat agree | Neither agree or disagree | Somewhat disagree | Strongly disagree |
| --- | --- | --- | --- | --- | --- |
| General Definition |  |  |  |  |  |
| Caveats |  |  |  |  |  |
| Examples |  |  |  |  |  |

**Do you agree with the following items?**

|  | Strongly agree | Somewhat agree | Neither agree or disagree | Somewhat disagree | Strongly disagree |
| --- | --- | --- | --- | --- | --- |
| General Definition |  |  |  |  |  |
| Caveats |  |  |  |  |  |
| Examples |  |  |  |  |  |

**In the box below, please enter any comments that you would like to add regarding this definition.**

| **Term** | 1. Reclining |
| --- | --- |
| **General Definition** | Reclining is a relaxed body position between sitting and lying. |
| **Caveats** | General definition applies to all age and ability groups. |
| **Examples** | **All age and ability groups:**Lounging/slouching on a chair or couch while sedentary. |
| **References** |  |

**Are the following items clearly stated?**

|  | Strongly agree | Somewhat agree | Neither agree or disagree | Somewhat disagree | Strongly disagree |
| --- | --- | --- | --- | --- | --- |
| General Definition |  |  |  |  |  |
| Caveats |  |  |  |  |  |
| Examples |  |  |  |  |  |

**Do you agree with the following items?**

|  | Strongly agree | Somewhat agree | Neither agree or disagree | Somewhat disagree | Strongly disagree |
| --- | --- | --- | --- | --- | --- |
| General Definition |  |  |  |  |  |
| Caveats |  |  |  |  |  |
| Examples |  |  |  |  |  |

**In the box below, please enter any comments that you would like to add regarding this definition.**

| **Term** | 1. Lying |
| --- | --- |
| **General Definition** | Lying refers to being in a horizontal position on a supporting surface.^1^ |
| **Caveats** | General definition applies to all age and ability groups. |
| **Examples** | **All age and ability groups:**Lying on a couch or bed. |
| **References** | ^1^ Concise Oxford English Dictionary. |

**Are the following items clearly stated?**

|  | Strongly agree | Somewhat agree | Neither agree or disagree | Somewhat disagree | Strongly disagree |
| --- | --- | --- | --- | --- | --- |
| General Definition |  |  |  |  |  |
| Caveats |  |  |  |  |  |
| Examples |  |  |  |  |  |

**Do you agree with the following items?**

|  | Strongly agree | Somewhat agree | Neither agree or disagree | Somewhat disagree | Strongly disagree |
| --- | --- | --- | --- | --- | --- |
| General Definition |  |  |  |  |  |
| Caveats |  |  |  |  |  |
| Examples |  |  |  |  |  |

**In the box below, please enter any comments that you would like to add regarding this definition.**

| Term | 1. Sedentary Behaviour Pattern |
| --- | --- |
| General Definition | The manner in which sedentary behaviour is accumulated throughout the day (e.g., the timing, duration and frequency of sedentary bouts and breaks).^1-2^ |
| Caveats | General definition applies to all age and ability groups. |
| Examples | - Prolonger: Someone who accumulates sedentary time in extended continuous bouts.^3^ - Breaker: Someone who accumulates sedentary time with frequent interruptions and in short bouts.^3^ |
| References | ^1^ Chinapaw MJ, de Niet M, Verloigne M, De Bourdeaudhuij I, Brug J, Altenburg TM. From sedentary time to sedentary patterns: accelerometer data reduction decisions in youth. PLoS One. 2014;9(11):e111205. ^2^ Chastin SF, Granat MH. Methods for objective measure, quantification and analysis of sedentary behaviour and inactivity. Gait Posture. 2010 Jan;31(1):82-6.  ^3^ Dunstan DW, Healy GM, Sugiyama T, Owen N. Too much sitting and metabolic risk — has modern technology caught up with us? Eur Endocrinol. 2010;6(1):19–23. |

**Are the following items clearly stated?**

|  | Strongly agree | Somewhat agree | Neither agree or disagree | Somewhat disagree | Strongly disagree |
| --- | --- | --- | --- | --- | --- |
| General Definition |  |  |  |  |  |
| Caveats |  |  |  |  |  |
| Examples |  |  |  |  |  |

**Do you agree with the following items?**

|  | Strongly agree | Somewhat agree | Neither agree or disagree | Somewhat disagree | Strongly disagree |
| --- | --- | --- | --- | --- | --- |
| General Definition |  |  |  |  |  |
| Caveats |  |  |  |  |  |
| Examples |  |  |  |  |  |

**In the box below, please enter any comments that you would like to add regarding this definition.**

These definitions along with the findings from this survey will be published in a peer-reviewed journal. In order to be an author on this publication, you must fully complete the survey, review the draft manuscript (to be circulated when prepared) and identify yourself so we can confirm your participation in this survey.

**Do you wish to be an author on the publication?**

|  | Yes |
| --- | --- |
|  | No |

**Full name**

**Phone number**

**Email address**

**Affiliation**

**Mailing Address**
